# Androgen receptor and FOXA1 coexpression define a “luminal-AR” subtype of feline mammary carcinomas, spontaneous models of breast cancer

**DOI:** 10.1186/s12885-019-6483-6

**Published:** 2019-12-30

**Authors:** Elie Dagher, Violette Royer, Paul Buchet, Jérôme Abadie, Delphine Loussouarn, Mario Campone, Frédérique Nguyen

**Affiliations:** 1 AMaROC, Oniris (Nantes Atlantic College of Veterinary Medicine, Food Science and Engineering), Oniris site Chantrerie, CS40706, 44307, Cedex 3, Nantes, France; 2 CRCINA, INSERM, Université d’Angers, Université de Nantes, Nantes, France; 3 Hôtel-Dieu CHU de Nantes, Anatomie Pathologique, cedex 01, 44093 Nantes, France; 4 Integrated Center for Oncology, ICO, 15 rue André Boquel, cedex 02, 49055 Angers, France

**Keywords:** Androgen receptor, Feline mammary carcinoma, FOXA1, Luminal-AR breast cancer, Spontaneous animal model, Triple-negative breast cancer

## Abstract

**Background:**

Invasive mammary carcinomas that spontaneously develop in female cats are associated with high mortality, and resemble the most aggressive human breast cancers, especially triple-negative breast cancer (TNBC). Transcriptome studies showed that TNBCs are a heterogeneous group that includes a potentially hormone-dependent subtype named luminal-AR. Some authors proposed an immunohistochemical definition of the luminal-AR subtype, which is not only positive for Androgen Receptor (AR), but also either positive for the transcription factor Forkhead box A1 (FOXA1), or negative for basal markers. The objectives of this study were to describe AR and FOXA1 expressions in feline mammary carcinomas (FMCs), their prognostic value, and if their coexpression could define a “luminal-AR” subtype of triple-negative mammary carcinomas in cats.

**Methods:**

In a previously described retrospective cohort of 180 female cats with FMCs, with a 2-year follow-up post-mastectomy, we assessed AR, FOXA1, ER, PR, Ki-67, HER2, and CK14 expressions by automated immunohistochemistry.

**Results:**

Of the 180 FMCs, 57 (32%) were luminal; i.e., ER and/or PR positive, and 123 (68%) were triple-negative (ER–, PR– and HER2–) FMCs. AR overexpression (found in 33 cases/180, 18%) and FOXA1 index ≥1% (64/180, 36%) were associated with a longer disease-free interval, overall survival, and cancer-specific survival in cats with FMC. Analysis of AR, FOXA1 and CK14 coexpression in triple-negative FMCs showed that AR+ triple-negative FMCs were heterogeneous: there existed an AR+ FOXA1+ CK14– subgroup (*n* = 7) associated with a better cancer-specific survival by multivariate survival analysis (HR = 0.26, 95% CI: 0.07–0.89, *p* = 0.03) compared to AR+ FOXA1–CK14+ triple-negative FMCs (*n* = 46) (HR = 1.00), independently of the pathologic tumor size and pathologic nodal stage. The non-basal-like subtype of triple-negative FMCs that coexpresses AR and FOXA1 (the AR+ FOXA1+ CK14– subgroup) could represent the equivalent of the luminal-AR subgroup of human triple-negative breast cancer.

**Conclusions:**

We identified an AR+ FOXA1+ CK14– subgroup of triple-negative FMCs that might correspond to the luminal-AR subgroup of human triple-negative breast cancers. Cats with FMC may be interesting spontaneous animal models to investigate new strategies targeting the androgen receptor, especially in the aggressive subtype of AR+ basal-like triple-negative mammary carcinomas with loss of FOXA1 expression (the AR+ FOXA1–CK14+ subgroup).

## Background

At least three factors justify that invasive mammary carcinomas that spontaneously arise in pet cats were proposed as relevant animal models for human breast cancer. The first is their high frequency: mammary tumors are reported to constitute 17% of neoplasms in female cats [1], are malignant in 80–90% of the cases [2, 3], and most of these malignant tumors are carcinomas. The second is their aggressive biological behavior: the median overall survival time of cats with invasive mammary carcinomas is 8–12 months post-diagnosis in most studies with follow-up [4–9]. The third is their resemblance with the most aggressive subtypes of breast cancer, for which targeted therapies are still needed: feline mammary carcinomas (FMCs) often lack significant levels of Estrogen Receptor (ER) and Progesterone Receptor (PR) expression, are rarely positive to HER2 (Human Epidermal growth factor Receptor 2), and thus most of them are considered to be triple-negative mammary carcinomas [10–19].

Transcriptome studies showed that triple-negative breast cancers (TNBCs) are a heterogeneous group. Among the major subtypes, the luminal-Androgen Receptor (luminal-AR) subtype appears to be a stable subgroup, characterized by high expression of downstream Androgen Receptor (AR) targets and coactivators [20]. Indeed, luminal-AR TNBC has been reported in multiple gene expression studies as reported by Lehmann et al. (2011 and 2016) [20, 21], Burstein et al. (2015) [22], and Jézéquel et al. (2015 and 2019) [23, 24]. Among others, luminal-AR breast cancers highly express the Forkhead box A1 (*FOXA1*) gene [20]. The transcription factor FOXA1 is able to bind to highly compacted chromatin, and acts as a pioneer factor with chromatin opening potential that allows other transcription factors and steroid hormone receptors to trigger transcriptional programs [25]. In the healthy mammary epithelial cell as well as in ER-positive breast cancers, FOXA1 is necessary for the expression of ERα target genes [25, 26]. In addition, preclinical studies suggest that FOXA1 allows AR to bind to DNA and thereby induce transcription of AR target genes and stimulate tumor proliferation [27].

Since transcriptome studies are expensive, some authors proposed an immunohistochemical definition of the luminal-AR subtype of triple-negative breast cancer. Guiu et al. (2015) hypothesized that these two markers, AR and FOXA1, are needed to identify the luminal-AR TNBC subtype by immunohistochemistry [28]. Other authors such as Astvatsaturyan et al. (2018) defined the luminal-AR subtype of TNBC by its positivity for AR and negativity for Epidermal Growth Factor Receptor (EGFR), a basal marker [29].

As most of feline invasive mammary carcinomas are triple-negative [11, 13, 19], the question arises of their heterogeneity. Here, we hypothesized that feline triple-negative mammary carcinomas may comprise, as in human TNBCs, a luminal-AR subtype, expected to be a non-basal-like TNBC with AR and FOXA1 coexpression, associated with a different biological behavior than other triple-negative FMCs.

The objectives of this study were thus 1) to investigate for the first time AR and FOXA1 expression in a large series of feline mammary carcinomas, 2) to assess their relationships with other clinical and pathological features of FMCs, 3) to investigate their prognostic significance in the feline patient, and 4) to put the first cornerstone for a better characterization of triple-negative FMCs, with identification of a luminal-AR subtype.

## Methods

### Animals and inclusion criteria

The present cohort of 180 female cats has been previously described [13]. Briefly, invasiveness of the included FMCs was confirmed by immunohistochemistry to a myoepithelial cell marker, p63 (clone 4A4, abcam ab111449), which can be used in both human [30, 31] and feline [32] mammary carcinomas. Feline patients benefited only from mastectomy. At diagnosis, clinical evaluation and medical imaging (radiography and/or ultrasonography) allowed excluding cases with another (non-mammary) malignancy, and to define the distant metastasis status: M0 (medical imaging revealed no distant metastasis), M1 (presence of distant metastases), or MX (no medical imaging performed). Follow-up was determined by veterinary evaluation for at least 48 months post-surgery. Prognosis was assessed in terms of disease-free interval (DFI), overall survival (OS), and cancer-specific survival (SS) [13]. This study was approved by the local ethical committee of our institution (CERVO, Comité d’Ethique en Recherche clinique et épidémiologique Vétérinaire d’Oniris, Nantes, France). Owner’s written consent to participate was obtained prior to inclusion.

### Histologic methods and criteria

Hematoxylin-Eosin-Saffron (HES)-stained sections of the FMCs were used to assess multicentricity, the histological type of feline mammary carcinoma, the pathologic tumor size (pT) in millimeters, presence/absence of lymphovascular invasion (LVI), central necrosis, squamous differentiation, and tumor-associated lymphohistiocytic inflammation, as previously described [13, 33]. The histological grade was assessed using Elston and Ellis criteria for human breast cancer [34], which have been validated in cats [33]. The pathologic nodal stage was defined as pN0 (negative pathologic nodal stage, on both an HES-stained section of the draining lymph node and an immunostained section for cytokeratins, clones AE1-AE3, Dako M3515), pN+ (positive pathologic nodal stage, for the presence of isolated tumor cells (< 0.2 mm in diameter), micro-metastases (0.2–2.0 mm in diameter), or macrometastases (> 2.0 mm) in the draining lymph node), or pNX (absence of lymph node sampled for histopathological examination). This allowed staging of the FMCs according to the modified World Health Organization (WHO) criteria [35, 36].

### Immunohistochemistry

The immunohistochemical expression of Estrogen Receptor alpha (ERα), Progesterone Receptor (PR), Human Epidermal Growth Factor Receptor 2 (HER2), the proliferation marker Ki-67, Cytokeratin 14 (CK14), Androgen Receptor (AR), and Forkhead box A1 (FOXA1) were assessed using a Benchmark XT stainer (Ventana Medical Systems, Roche Diagnostics) (Table 1). Antibodies to ER, PR, HER2, Ki-67 and CK14 have been previously validated in cats [13, 37]. Antibodies to AR and FOXA1 were used here for the first time in cats. According to the NCBI-BLAST website (https://blast.ncbi.nlm.nih.gov/Blast.cgi), amino acid identity is 87% between human AR and feline AR proteins, and 89% between human FOXA1 and feline FOXA1 proteins. Specificity of the anti-AR clone SP107 in cats was validated on normal feline tissues: AR immunoreactivity was found in the testis (interstitial Leydig cells, peritubular myoid cells), epididymis, uterus, ovary, hyperplastic mammary gland, cutaneous sebaceous glands, but absent in the gastrointestinal tract, liver, lung, heart, thyroid, parathyroid, hematopoietic and lymphoid tissues, skeletal muscle, adipose tissue, and nervous system, as expected in humans (https://www.proteinatlas.org). Similarly, the anti-FOXA1 clone SP88 cross-reacted with feline FOXA1, and was considered specific, as immunoreactivity was restricted to the hyperplastic mammary gland, cutaneous sweat glands, oviduct, bronchial epithelium, urinary bladder urothelium, and rare lymphocytes, as described in humans (https://www.proteinatlas.org).

**Table 1:** Immunohistochemical protocols

Antigen	Clone and origin	Dilution, incubation time	Source, reference	Antigen retrieval	Detection system
ERα	C311 mouse mAb	1:50 44 min	Santa Cruz Biotechnology, sc-787	None	iView DAB detection kit (Ventana Medical Systems, Roche Diagnostics, 760–091)
PR	10A9 mouse mAb	1:50 1 h 40 min.	Meridian Life Science, K42546 M	HIER, CC1, 1 h	iView DAB detection kit
HER2	4B5 rabbit mAb	Prediluted 8 min	Roche Diagnostics, 790–2991	HIER, CC1, 30 min	UltraView Universal DAB detection kit (Ventana Medical Systems, Roche Diagnostics, 760–500)
Ki-67	MIB1 mouse mAb	1:50 32 min	Dako, M7240	HIER, CC1, 1 h	iView DAB detection kit
Cytokeratin 14	LL002 mouse mAb	1:150 44 min	Santa Cruz Biotechnology, sc-58,724	HIER, CC1, 56 min	iView DAB detection kit
AR	SP107 rabbit mAb	1:400 1 h 20 min.	Spring, M4070	HIER, CC1, 30 min	OptiView DAB IHC Detection Kit (Ventana Medical Systems, Roche Diagnostics, 760–700)
FOXA1	SP88 rabbit mAb	1:50 1 h 20 min.	Spring, M3884	HIER, CC1, 56 min	OptiView DAB IHC Detection Kit

*CC1* Cell Conditioning solution 1, Ventana Medical Systems (reference 950–124)

*HIER* Heat-induced epitope retrieval

*mAb* monoclonal antibody

For negative controls, the primary antibodies were replaced by normal rabbit or mouse sera (prediluted reagents, Roche Diagnostics). Positive internal controls were the peritumoral mammary gland for ERα, PR, AR and FOXA1, cutaneous sebaceous glands for ERα and AR, the epidermis and hair follicles for Ki-67 and CK14, and sweat glands for FOXA1. For HER2 IHC, the pathway HER2 4-in-1 control slides (Roche Diagnostics) were used as external positive controls.

A medical doctor specialist in breast cancer pathology (DL) and three certified veterinary pathologists (JA, FN, ED), blinded to the clinical outcome or clinicopathologic data, evaluated the immunostochemical data. ERα and PR were considered positive at a 10% threshold, as previously reported for dogs [38–40] and cats [13] with mammary carcinomas, and human breast cancers [41]. A threshold of 20% for the Ki-67 index was used to differentiate highly and poorly proliferative FMCs among hormone receptor-positive cases [42]. HER2 scores were assigned according to the recommendations for HER2 testing by IHC in breast cancers [43]. CK14 was considered positive when more than 15% of the tumor cells expressed the protein in their cytoplasm.

The 180 invasive feline mammary carcinomas were classified as luminal (ER+ and/or PR+, any HER2 score) or triple-negative (ERα < 10%, PR < 10%, HER2 score 0 to 2+), including basal-like triple-negative carcinomas (ERα < 10%, PR < 10%, HER2 score 0 to 2+, CK14 ≥ 15%), as previously described [10, 13, 18, 19].

Nuclear AR expression was quantified as an index (percentage of positive neoplastic cells), and as an Allred score, whereas cytoplasmic AR immunoreactivity was not considered in scoring. The Allred score is the sum of a proportion score, reflecting the percentage of AR-positive cells by immunohistochemistry (nuclear signal, on a scale of 0 to 5 points, respectively for 0, ≤1%, 1–10%, 11–33%, 34–66% and ≥ 67% of AR+ cells), and an intensity score (on a scale of 0 to 3 points, respectively for negative, weak, intermediate, and strong staining), for a possible total score of 8 points. AR overexpression was defined by Allred scores of 7–8 points, and AR positivity was defined as AR index ≥25%. FOXA1 expression was quantified as the percentage of positive neoplastic cells (with nuclear signal) in at least 500 cancer cells (FOXA1 index in %). The prognostic cutoffs (1% for FOXA1, 25% for AR positivity, Allred scores 7–8 for AR overexpression) were determined by receiver-operating-characteristic curve analyses calculated for 2-year cancer-specific survival.

### Statistical analyses

We have used the MedCalc® statistical software (Ostend, Belgium) for all of the statistical analyses. Statistical associations between the clinicopathologic characteristics were evaluated using Chi-2 tests for categorical variables, one-way analysis of variance between a continuous and a categorical variable, and linear regression analysis among continuous variables. Univariate survival analyses were performed using the Kaplan-Meier method and log-rank tests, while multivariate survival analyses relied on Cox proportional hazards models. The results are expressed as the Hazard Ratio (HR), its 95% confidence interval (95% CI), and the *p*-value of each covariate. The significance threshold was set at < 0.05.

## Results

### Cohort description

The main characteristics of the 180 female cats are reported in Table 2. The mammary carcinoma was diagnosed at a mean age of 11.1 ± 2.7 years (range, 4.0–19.3 years). The cats were mainly European shorthair or longhaired cats (154/180, 86%), and Siamese (15/180, 8%).

**Table 2:** Patients characteristics

Parameters	Categories	N	%
Breed	European shorthair or longhair	154	85.6%
	Other breeds	26	14.4%
Gender	Intact female	112	62.2%
	Neutered female	68	37.8%
History of contraception	Yes	76	42.2%
	No	18	10.0%
	Unknown	86	47.8%
Previous benign mammary lesions	Yes	16	8.9%
	No	164	91.1%
Parity	Nulliparous	15	8.3%
	Multiparous	21	11.7%
	Unknown	144	80.0%
Multicentricity	Multiple FMCs	26	14.4%
	Single FMC	154	85.6%
Pathologic tumor size	pT < 20 mm	85	47.2%
	pT ≥ 20 mm	95	52.8%
Pathologic nodal stage	pN+ (nodal metastasis)	101	56.1%
	pN0 (no)	20	11.1%
	pNX (unknown)	59	32.8%
Distant metastasis	M1 (yes)	8	4.4%
	M0 (no)	64	35.6%
	MX (unknown)	108	60.0%
WHO stage	Stage I	45	25.0%
	Stage II	23	12.8%
	Stage III	104	57.8%
	Stage IV	8	4.4%
WHO Histological type	Cribriform	54	30.0%
	Comedocarcinoma	50	27.8%
	Solid	27	15.0%
	Mucinous	15	8.3%
	Tubulopapillary	12	6.7%
	Tubular	9	5.0%
	Papillary	7	3.9%
	Adenosquamous	4	2.2%
	Anaplastic	2	1.1%
Elston and Ellis histological grade	Grade I	10	5.5%
	Grade II	82	45.6%
	Grade III	88	48.9%
Lymphovascular invasion	LVI+	110	61.1%
	LVI–	70	38.9%
Tumor-associated inflammation	Absent to mild	77	42.8%
	Moderate to severe	103	57.2%
Estrogen Receptor (ERα)	ER+ (ER ≥ 10%)	49	27.2%
	ER– (ER < 10%)	131	72.8%
Progesterone Receptor (PR)	PR+ (PR ≥ 10%)	13	7.2%
	PR– (PR < 10%)	167	92.8%
HER2 Score	0	103	57.2%
	1+	59	32.8%
	2+	18	10.0%
	3+	0	0
Ki-67	High Ki-67 (≥ 20%)	169	93.9%
	Low Ki-67 (< 20%)	11	6.1%
CK14	CK14+ (≥ 15%)	132	73.3%
	CK14– (< 15%)	48	26.7%

*WHO* World Health Organization

The pT (mean, 18 ± 7 mm; median, 18 mm; range, 4–48 mm) was measurable in 150 cases, and imprecise in the 30 remaining cases, due to positive tumor margins. A hundred and one patients (56%) had a positive pathologic nodal stage (pN+), and 8 (4%) had distant metastases (M1) at diagnosis. The 180 FMCs were diagnosed at stage I in 45 cats (25%), stage II in 23 cats (13%), stage III in 104 cats (58%) and stage IV in 8 cats (4%).

Central necrosis was present in 160 cases (89%), squamous differentiation in 81 cases (45%), lymphovascular invasion (LVI) in 110 cases (61%), and moderate to severe tumor-associated inflammation in 103 cases (57%). According to the Elston and Ellis grading system, 10 cases (5%) were grade I, 82 (46%) were grade II and 88 (49%) were grade III FMCs.

The mean ERα index was 10.0 ± 13.3% (median, 5.4%; range, 0–74.2%), with 88% of cases (158/180) showing at least one ER+ neoplastic cell, but only 49 FMCs (27%) were ER-positive. The mean PR index was 3.0 ± 11.0% (median, 0%; range, 0–87.8%), most FMCs (144/180, 80%) were totally devoid of PR expression, and only 13 (7%) were PR-positive. None of the carcinomas overexpressed HER2 (3+ immunohistochemical score). The mean Ki-67 index was 45 ± 14% (median, 45%; range, 13–83%). Fifty-seven FMCs (32%) were luminal (ER- and/or PR-positive, any HER2 score), including 8 luminal-A (Ki-67 index < 20%) and 49 luminal-B (Ki-67 ≥ 20%) FMCs, and 123 (68%) were triple-negative (ER–, PR–, HER2–).

At 15% threshold for CK14 positivity, 132/180 (73%) FMCs were CK14+, including 39 (68%) of the 57 luminal FMCs and 93 (76%) of the 123 triple-negative FMCs.

### AR expression in FMCs

In the mammary gland surrounding FMCs, nuclear AR expression was usually intense but patchy, restricted to luminal cells in hyperplastic lobules and ducts; this level of expression corresponded to Allred scores ranging from 2 (weak staining in ≤1% of mammary epithelial cells) to 6 (strong AR expression in less than one-third of mammary epithelial cells). In FMCs, positive immunohistochemical staining for AR was most commonly nuclear, and was observed in neoplastic cells as well as scarce stromal cells including endothelial cells and cancer-associated fibroblasts (data not shown). Cytoplasmic AR immunoreactivity was observed in 82 FMCs (46%), but was not taken into account for AR scoring. The mean percentage of AR-positive neoplastic cells (AR index) was 45 ± 25% (median 45%, range 0–95%). Most of the carcinomas (174/180, 97%) contained at least one AR-positive neoplastic cell, whereas only few FMCs (6/180, 3%) were completely devoid of AR expression. At 10% threshold for AR positivity, 92% of the cases (165/180) were positive for AR. When AR expression was quantified as an Allred Score, 33/180 (18%) FMCs overexpressed AR (Allred scores 7–8 points), including 12/57 (21%) luminal FMCs and 20/123 (16%) triple-negative FMCs.

AR overexpression (Allred score ≥ 7) was negatively correlated with (1) pathologic nodal stage (OR = 0.32, 95% CI: 0.14–0.71, *p* < 0.001): only 33% (11/33) of FMCs with AR overexpression were pN+ compared to 61% (90/147) of AR-negative FMCs; (2) lymphovascular invasion (OR = 0.28, 95% CI: 0.13–0.62, *p* < 0.01): only 37% (12/33) of FMCs with AR overexpression were LVI+ compared to 67% (99/147) of AR-negative FMCs, and (3) clinical stage at diagnosis (OR = 0.32, 95% CI: 0.15–0.70 p < 0.01): only 41% (13/33) of FMCs with AR overexpression were diagnosed at stage III or IV compared to 67% (99/147) of AR-negative FMCs. In addition, the AR index was negatively associated with (4) the pathologic tumor size (R^2^ = 0.026 and *p* = 0.048), (5) the Elston and Ellis histological grade (*p* = 0.002): grade I and II FMCs had an AR index of 51 ± 24% compared to 39 ± 24% for grade III carcinomas, and (6) tumor-associated inflammation (*p* = 0.016): FMCs with moderate to severe tumor-associated inflammation had an AR index of 41 ± 25%, compared to 50 ± 25% in FMCs with absent to mild tumor-associated inflammation.

In triple-negative FMCs, AR overexpression was negatively correlated with (1) the Elston and Ellis histological grade (OR = 0.25, 95% CI: 0.08–0.80, *p* = 0.020): only 20% (4/20) of AR-overexpressing triple-negative FMCs were grade III compared to 50% (51/103) of AR-negative triple-negative FMCs, and (2) lymphovascular invasion (OR = 0.33, 95% CI: 0.12–0.88, *p* = 0.040): only 40% (8/20) of AR-overexpressing triple-negative FMCs were LVI+ compared to 67% (69/103) of AR-negative triple-negative FMCs.

### FOXA1 expression in FMCs

Positive immunohistochemical staining to FOXA1 was also nuclear, and was observed in neoplastic cells as well as scarce tumor-infiltrating lymphocytes (data not shown). In the 180 FMCs, the mean FOXA1 index was 3.8 ± 9.9% (median 0, range 0–85.1%). Seventy-eight (43%) of the carcinomas contained at least one FOXA1-positive neoplastic cell whereas the other 102 (57%) were completely devoid of FOXA1 expression. At a FOXA1 index ≥1%, 64/180 (36%) FMCs were FOXA1+, including 28/57 (49%) luminal FMCs and 36/123 (29%) triple-negative FMCs.

FOXA1 index ≥1% was negatively correlated with the (1) pathologic nodal stage (OR = 0.46, 95% CI: 0.25–0.46, *p* = 0.020): only 44% (28/64) of FOXA1-positive FMCs were pN+ compared to 63% (73/116) of FOXA1-negative FMCs, (2) Elston and Ellis histological grade (OR = 0.14, 95% CI: 0.03–0.70, *p* = 0.010): 89% (57/64) of FOXA1-positive FMCs were grade II or III compared to 98% (114/116) of FOXA1-negative FMCs, (3) clinical stage at diagnosis (OR = 0.37, 95% CI: 0.20–0.70, *p* = 0.003): 47% (30/64) of FOXA1-positive FMCs were stage III or IV compared to 71% (82/116) of FOXA1-negative FMCs, (4) lymphovascular invasion (OR = 0.34, 95% CI: 0.18–0.64, *p* < 0.001): only 44% (28/64) of FOXA1-positive FMCs were LVI+ compared to 70% (81/116) of FOXA1 negative FMCs, and (5) the Ki-67 proliferation index (OR = 0.51, 95% CI: 0.27–0.95, *p* = 0.040): 50% (32/64) of FOXA1-positive FMCs had a high proliferation index (≥ 42%) compared to 66% (77/116) of FOXA1-negative FMCs. FOXA1 index ≥1% was positively correlated to (6) PR positivity (OR = 7.74, 95% CI: 3.42–17.51, *p* < 0.0001): 42% (27/64) of FOXA1-positive FMCs were PR+ compared to only 9% (10/116) of FOXA1-negative FMCs, (7) AR (*p* = 0.032): FOXA1-positive FMCs had an AR index of 50 ± 25% compared to 42 ± 25% in FOXA1-negative FMCs, and finally FOXA1 was positively correlated with (8) the luminal phenotype (OR = 2.33, 95% CI: 1.22–4.46, *p* = 0.010): 44% (28/64) of FOXA1 positive FMCs were luminal compared to 25% (29/116) of FOXA1 negative FMCs.

In triple-negative FMCs, FOXA1 index ≥1% was negatively correlated with (1) lymphovascular invasion (OR = 0.40, 95% CI: 0.18–0.89, *p* = 0.040): only 47% (17/36) of FOXA1-positive triple-negative FMCs were LVI+, compared to 69% (60/87) of FOXA1-negative triple-negative FMCs. The FOXA1 index of was also negatively correlated to (2) clinical stage (*p* = 0.026): stage I–II triple-negative FMCs had a FOXA1 index of 2.7 ± 6.2% compared to only 0.9 ± 2.4% for stage III–IV triple-negative carcinomas, (3) pathologic nodal stage (*p* = 0.049): the 70 pN+ triple-negative FMCs had a FOXA1 index of only 0.9 ± 2.6%, compared to 2.5 ± 5.7% in the 53 pN0-pNX triple-negative carcinomas, (4) Elston and Ellis histological grade (*p* = 0.046): grade I–II triple-negative FMCs had a FOXA1 index of 2.3 ± 5.3%, compared to only 0.7 ± 2.0% for grade III triple-negative carcinomas, and finally FOXA1 expression was also positively correlated with (5) the AR index (R^2^ = 0.044 and *p* = 0.019).

### Prognostic value of AR and FOXA1 in FMCs

**Fig. 1:**
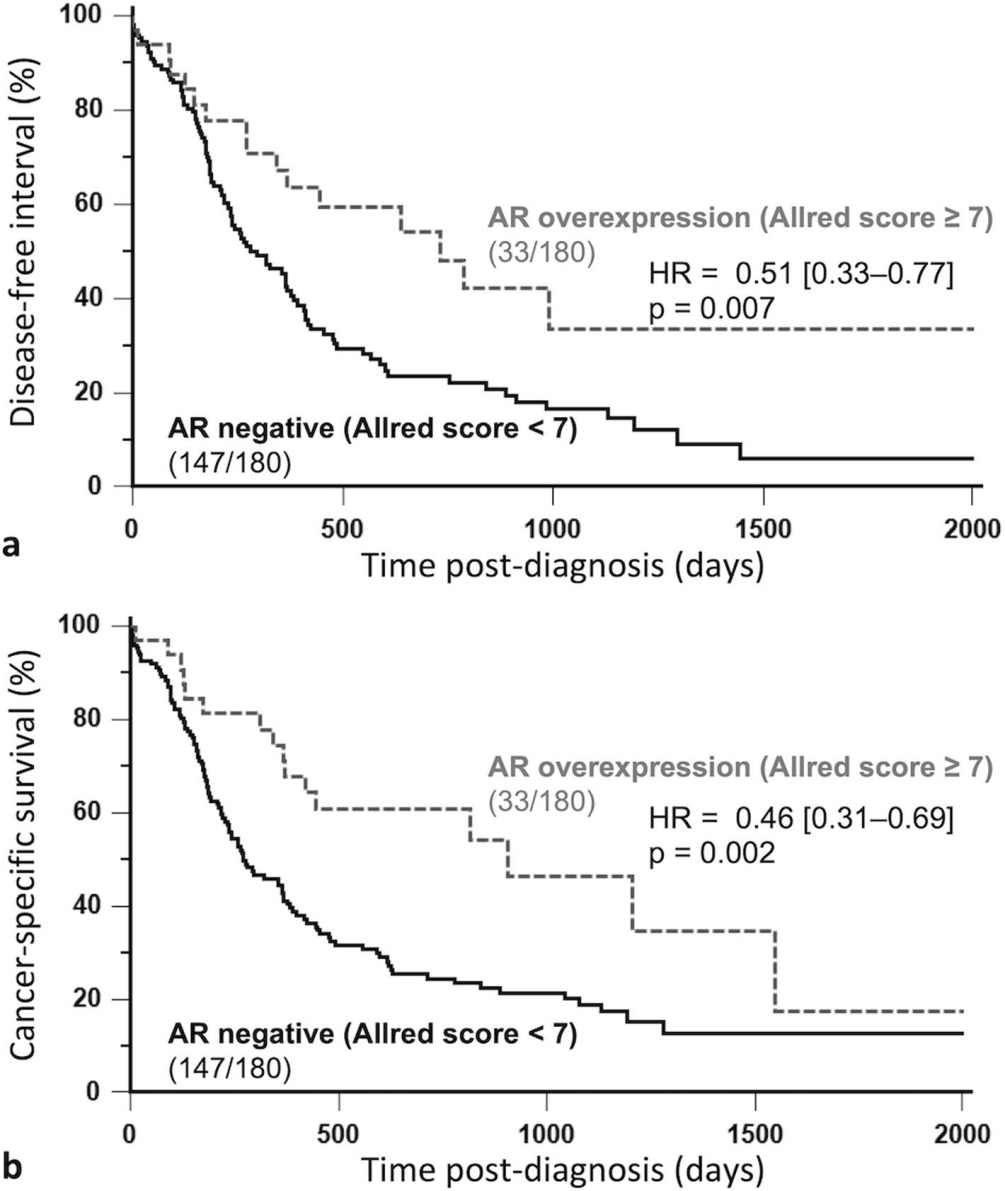
Kaplan-Meier survival curves of 180 cats according to AR overexpression (Allred score ≥ 7) in invasive mammary carcinomas. Disease-free interval (**a**), Cancer-specific survival (**b**). AR overexpression in feline mammary carcinomas was associated with better prognosis

AR overexpression (Allred score ≥ 7) was associated with longer disease-free interval (HR = 0.51, 95% CI: 0.33–0.77, *p* = 0.007; Fig. 1a) by univariate survival analysis. The other clinicopathologic factors significantly associated with disease-free interval were the pathologic tumor size (pT ≥ 20 mm versus < 20 mm: *p* = 0.025, HR = 1.51), pathologic nodal stage (pN+ versus pN0–pNX: *p* = 0.015, HR = 1.56), distant metastasis (M1 versus M0–MX: *p* < 0.0001, HR = 5.35), lymphovascular invasion (LVI+ versus LVI–: *p* = 0.0002, HR = 1.97), and PR positivity (PR ≥ 10% versus PR < 10%: *p* = 0.043, HR = 0.48). By multivariate survival analysis, AR overexpression was associated with longer disease-free interval, independently of the pathologic tumor size and distant metastasis (Table 3).

**Table 3:** Prognostic value of AR overexpression in FMCs estimated by multivariate analysis (180 cases)

	Categories	p	HR	95% CI
Disease-free interval				
AR overexpression	AR+ (Allred Score ≥ 7) vs. AR–	0.023	0.54	0.31–0.91
Pathologic tumor size	pT ≥ 20 mm versus < 20 mm	0.049	1.46	1.00–2.12
Distant metastasis	M1 versus M0–MX	< 0.0001	4.87	2.32–10.23
Overall survival				
AR overexpression	AR+ (Allred Score ≥ 7) vs. AR–	0.027	0.62	0.41–0.94
Pathologic tumor size	pT ≥ 20 mm versus < 20 mm	0.001	1.68	1.23–2.29
Distant metastasis	M1 versus M0–MX	0.007	2.72	1.32–5.64
Cancer-specific survival				
AR overexpression	AR+ (Allred Score ≥ 7) vs. AR–	0.024	0.54	0.32–0.92
Pathologic tumor size	pT ≥ 20 mm versus < 20 mm	0.004	1.69	1.18–2.42
Pathologic nodal stage	pN+ versus pN0–pNX	0.001	1.84	1.27–2.67
Distant metastasis	M1 versus M0–MX	0.002	3.23	1.55–6.76

AR overexpression (Allred score ≥ 7) was also associated with longer overall survival (HR = 0.60, 95% CI 0.42–0.85, *p* = 0.011) by univariate survival analysis, and the other clinicopathologic parameters associated with overall survival were patient age at diagnosis (*p* = 0.035, HR = 0.72 if ≤11 years), the pathologic tumor size (*p* = 0.0007, HR = 1.68 if pT ≥ 20 mm), pathologic nodal stage (*p* = 0.0001, HR = 1.78 if pN+), distant metastasis (*p* = 0.002, HR = 2.89 if M1), lymphovascular invasion (*p* < 0.0001, HR = 2.33 if LVI+), histological grade (*p* = 0.023, HR = 1.41 if grade III), cutaneous ulceration (*p* = 0.025, HR = 1.51 if present), tumor-associated inflammation (*p* = 0.0053, HR = 1.54 if moderate to severe), and PR positivity (*p* = 0.021, HR = 0.49 if PR ≥ 10%). By multivariate survival analysis, AR overexpression was associated with longer overall survival, independently of the pathologic tumor size and distant metastasis (Table 3).

Finally, AR overexpression (Allred score ≥ 7) was associated with longer cancer-specific survival (HR = 0.46, 95% CI: 0.31–0.69, *p* = 0.002, Fig. 1b) by univariate survival analysis. The other clinicopathologic factors associated with cancer-specific survival were the pathologic tumor size (*p* = 0.0006, HR = 1.82 if pT ≥ 20 mm), pathologic nodal stage (*p* = 0.0001, HR = 2.03 if pN+), distant metastasis (*p* = 0.0003, HR = 3.40 if M1), lymphovascular invasion (*p* < 0.0001, HR = 2.86 if LVI+), tumor-associated inflammation (*p* = 0.010, HR = 1.59 if moderate to severe), and PR positivity (p = 0.002, HR = 0.25 if PR ≥ 10%). The favorable prognostic value of AR overexpression in terms of cancer-specific survival was confirmed by multivariate analysis independently of the pathologic tumor size, pathologic nodal stage and distant metastasis (Table 3).

In luminal FMCs as well, AR overexpression was associated with favorable outcome in terms of cancer-specific survival (HR = 0.38, 95% CI: 0.19–0.74; *p* = 0.017) and overall survival (HR = 0.46, 95% CI: 0.25–0.82, *p* = 0.024). In triple-negative FMCs also, AR overexpression was associated with longer disease-free interval (HR = 0.49, 95% CI: 0.28–0.85; *p* = 0.037) and cancer-specific survival (HR = 0.53, 95% CI: 0.32–0.89; *p* = 0.046).

**Fig. 2:**
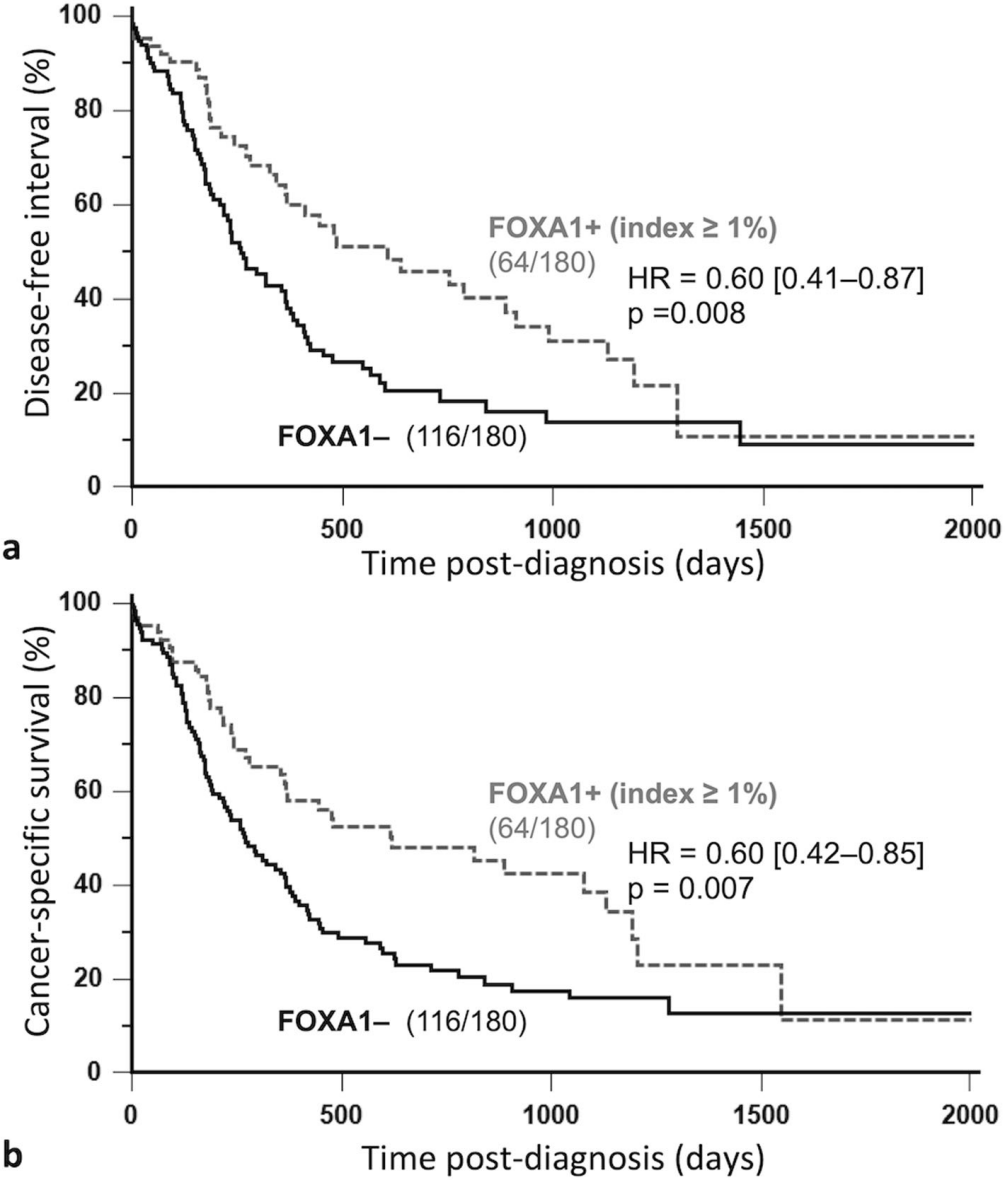
Kaplan-Meier survival curves of 180 cats according to FOXA1 positivity (index ≥1%) in invasive mammary carcinomas. Disease-free interval (**a**), Cancer-specific survival (**b**). FOXA1 index ≥1% in feline mammary carcinomas was associated with better prognosis

In the 180 FMCs studied, FOXA1 index ≥1% was associated with longer disease-free interval (HR = 0.60, 95% CI: 0.41–0.87, *p* = 0.008; Fig. 2a), longer overall survival (HR = 0.73, 95% CI: 0.53–0.99, *p* = 0.049), and longer cancer-specific survival (HR = 0.60, 95% CI: 0.42–0.85; *p* = 0.007; Fig. 2b) by univariate survival analysis. FOXA1 positivity (index ≥1%) was also associated with favorable outcome in luminal FMCs, in terms of disease-free interval (HR = 0.39, 95% CI: 0.21–0.75; *p* = 0.002) and cancer-specific survival (HR = 0.46, 95%-CI: 0.24–0.87; *p* = 0.014). However, FOXA1 index ≥1% was not significantly associated with outcome in triple-negative carcinomas.

By multivariate survival analysis, FOXA1 index ≥1% was associated with longer disease-free interval and overall survival independently of the pathologic tumor size and distant metastasis (Table 4). The favorable prognostic value of FOXA1 index ≥1% was also observed in cancer-specific survival independently of the pathologic tumor size, pathologic nodal stage and distant metastasis (Table 4).

**Table 4:** Prognostic value of FOXA1 index ≥1% in FMCs estimated by multivariate analysis (180 cases)

	Categories	p	HR	95% CI
Disease-free interval				
FOXA1 positivity	FOXA1 ≥ 1% versus < 1%	0.009	0.58	0.39–0.87
Pathologic tumor size	pT ≥ 20 mm versus < 20 mm	0.047	1.46	1.01–2.12
Distant metastasis	M1 versus M0–MX	< 0.0001	5.05	2.41–10.56
Overall survival				
FOXA1 positivity	FOXA1 ≥ 1% versus < 1%	0.030	0.70	0.50–0.96
Pathologic tumor size	pT ≥ 20 mm versus < 20 mm	0.001	1.69	1.24–2.30
Distant metastasis	M1 versus M0–MX	0.005	2.82	1.36–5.82
Cancer-specific survival				
FOXA1 positivity	FOXA1 ≥ 1% versus < 1%	0.032	0.65	0.44–0.96
Pathologic tumor size	pT ≥ 20 mm versus < 20 mm	0.004	1.69	1.18–2.42
Pathologic nodal stage	pN+ versus pN0–pNX	0.002	1.83	1.26–2.66
Distant metastasis	M1 versus M0–MX	0.001	3.47	1.66–7.23

### Diversity of AR-positive triple-negative FMCs

**Fig. 3:**
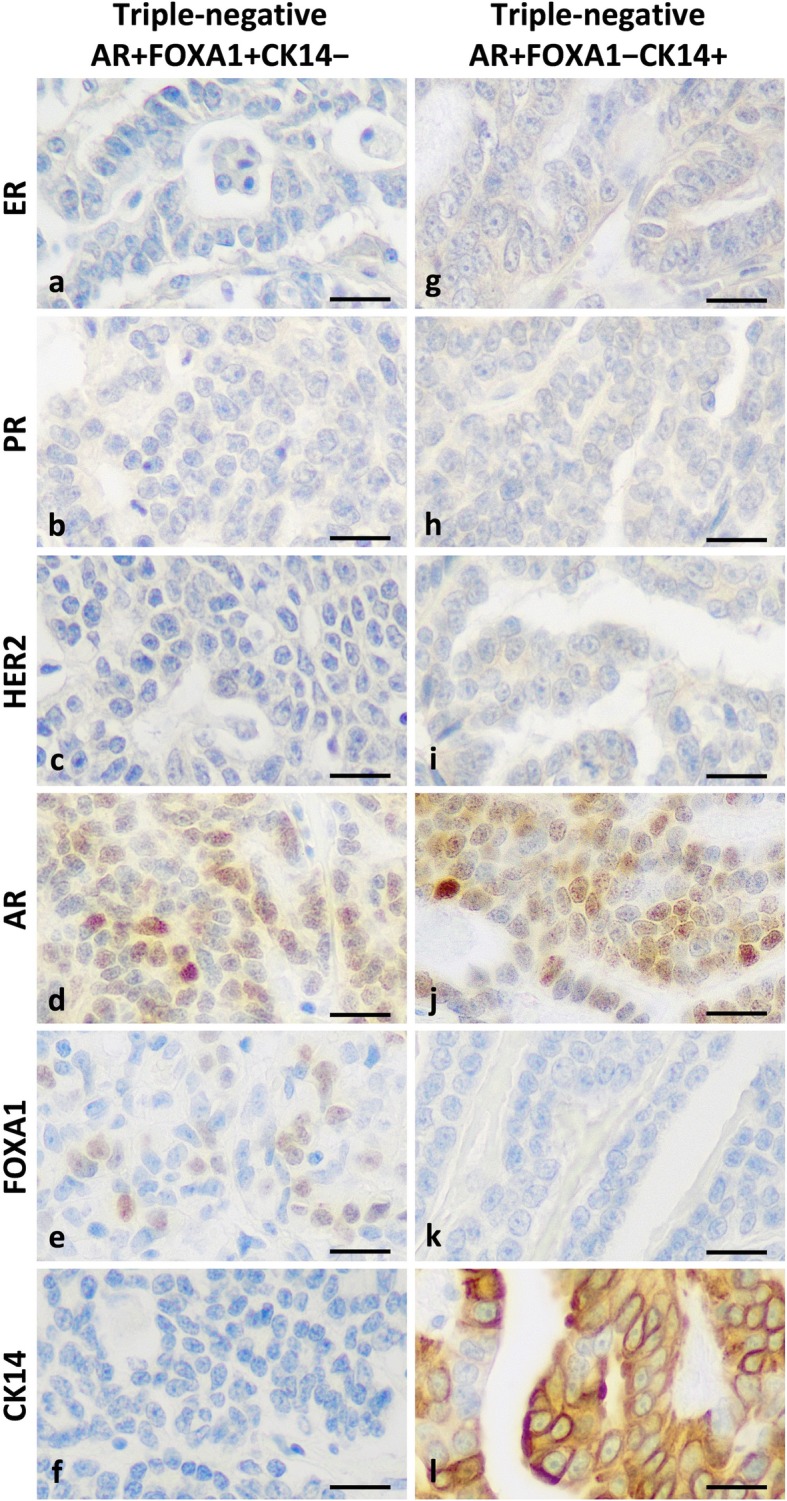
Diversity of AR-positive triple-negative feline mammary carcinomas. Left panel, non-basal-like AR+ FOXA1+ CK14– triple-negative feline invasive mammary carcinomas determined by immunohistochemistry were characterized by: ER negativity (**a**), PR negativity (**b**), HER2 negativity (**c**), AR positivity (**d**), FOXA1 positivity (**e**), and CK14 negativity (**f**). Right panel, basal-like AR+ FOXA1– CK14+ triple-negative feline invasive mammary carcinomas determined by immunohistochemistry were characterized by: ER negativity (**g**), PR negativity (**h**), HER2 negativity (**i**), AR positivity (**j**), FOXA1 negativity (**k**), and CK14 positivity (**l**). Peroxidase-DAB revelation system. Original magnification 400x. Bar = 20 μm

**Fig. 4:**
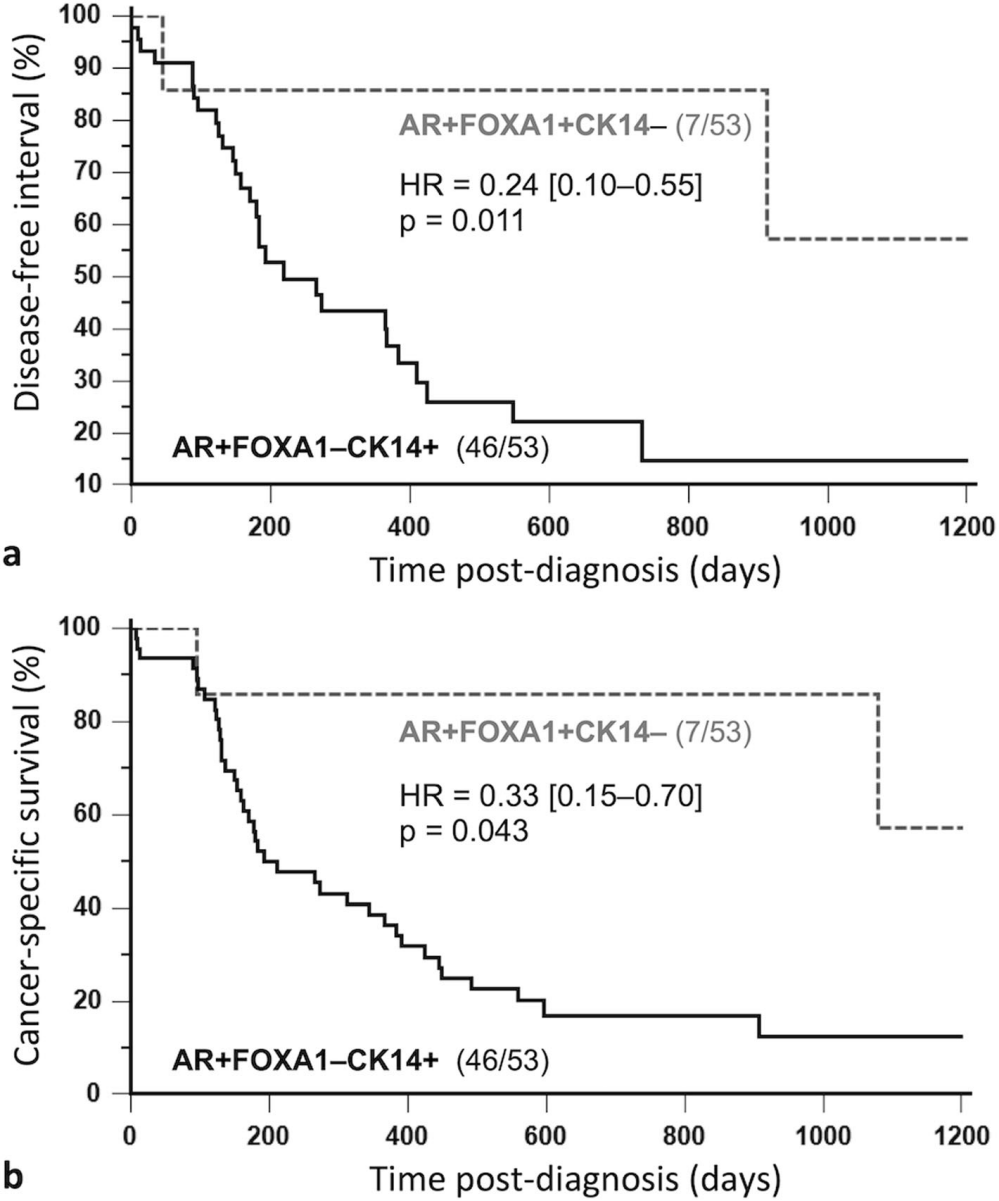
Kaplan-Meier survival curves of 53 cats with AR+ triple-negative mammary carcinomas according to FOXA1/CK14 status. Disease-free interval (**a**), Cancer-specific survival (**b**). Within AR+ triple-negative mammary carcinomas, the FOXA1+ CK14– phenotype was associated with much better prognosis than the FOXA1–CK14+ phenotype (i.e., loss of FOXA1 expression and basal-like subtype)

Of the 123 triple-negative FMCs, 53 (43%) were AR-positive (AR index ≥25%). Among AR-positive triple-negative FMCs, we identified two subgroups, a non-basal-like subgroup (Fig. 3 a–f; *N* = 7) characterized by double positivity to AR and FOXA1 (AR+ FOXA1+ CK14–), defined by AR positivity (AR index ≥25%), FOXA1 positivity (index ≥1%) and CK14 negativity (CK14 <  15%), and a basal-like subgroup (Fig. 3 g–l; *N* = 46) with loss of FOXA1 expression (AR+ FOXA1– CK14+). Both subgroups did not significantly differ by tumor stage, nodal status, stage at diagnosis, or histological grade (data not shown). However, the non basal-like AR+ FOXA1+ CK14– subgroup was associated with longer disease-free interval (*p* = 0.011, Fig. 4a) and longer cancer-specific survival (HR = 0.33, 95% CI: 0.15–0.70, *p* = 0.043; Fig. 4b) by univariate survival analysis compared to the basal-like AR+ FOXA1– CK14+ subgroup that was associated with a worse outcome.

By multivariate survival analysis, the non basal-like AR+ FOXA1+ CK14– subgroup was associated with longer cancer-specific survival (HR = 0.26, 95% CI: 0.07–0.89, *p* = 0.033) compared to the basal-like AR+ FOXA1– CK14+ subgroup (HR = 1.00) independently of the pathologic tumor size (pT ≥ 20 mm: HR = 2.15, 95% CI: 1.10–4.19, *p* = 0.026) and pathologic nodal stage (pN+: HR = 2.28, 95% CI: 1.17–4.46, *p* = 0.017) (Table 5). These results suggest that loss of FOXA1 expression and basal marker CK14 expression in triple-negative FMCs split the AR+ phenotype into two subgroups of different aggressiveness, a good-prognosis non basal-like AR+ FOXA1+ CK14– subgroup, and a poor-prognosis basal-like AR+ FOXA1–CK14+ subgroup.

**Table 5:** Prognostic value of FOXA1 and CK14 association in AR+ triple-negative FMCs (multivariate analysis, 53 cases)

Cancer-specific survival	Categories	p	HR	95% CI
AR/FOXA1/CK14 status	AR+ FOXA1+ CK14– versus AR+ FOXA1–CK14+	0.033	0.26	0.07–0.89
Pathologic tumor size	pT ≥ 20 mm versus < 20 mm	0.026	2.15	1.10–4.19
Pathologic nodal stage	pN+ versus pN0–pNX	0.017	2.28	1.16–4.46

## Discussion

This study investigates for the first time AR and FOXA1 expressions in a large cohort of feline invasive mammary carcinomas. The first objective was thus to determine if AR and FOXA1 expressions are common in FMCs. Most of the carcinomas (97%) contained AR-positive neoplastic cells. At 10% threshold for AR positivity, 92% of the cases would be considered positive for AR. In women, the frequency of AR expression in invasive ductal mammary carcinomas varies considerably depending on the studies and the thresholds used [44–51]. At the frequently used 10% threshold for AR positivity, from 58% [51] to 90% [46] of breast cancers are AR+, compared to 92% in cats with FMCs. Cats tend thus to have a higher AR expression in FMCs than women with breast cancer.

In our cohort, 43% of the carcinomas contained FOXA1-positive neoplastic cells. At FOXA1 index ≥1, 36% of the FMCs were considered positive for FOXA1. The prognostic cutoff for FOXA1 used in breast cancer varies considerably [52–55], reaching 71% for hormone receptor-positive breast cancers [52]. The frequency of FOXA1 positivity in breast cancer ranges from 41.5 to 85.9% of cases [52, 56–62]. Thus FOXA1 expression in breast cancer seems to be considerably higher than in FMCs. This difference may partly be due to the fact that most of the FMCs are triple-negative carcinomas, whereas FOXA1 is strongly associated with the luminal phenotype in women [57, 59].

The second objective of this study was to assess the relationships between AR, FOXA1, and the other clinical and pathological features of feline invasive mammary carcinomas. In the present study, there was a negative correlation between AR overexpression and pathologic tumor size, in agreement with results reported in human breast cancer [50, 51]. For instance, Ogawa et al. (2008) reported that 14% (19/134) of AR-positive breast cancers had a tumor size > 3 cm compared to 26% (21/81) of AR-negative carcinomas [50]. We also found a negative correlation between AR overexpression and lymphovascular invasion, including in the triple-negative subcohort. However, according to Tsang et al. (2014) in a large cohort of 1144 patients with primary invasive breast cancer, there is no significant correlation between AR positivity and lymphovascular invasion in human breast cancer [63]. We observed a negative correlation between AR expression and pathologic nodal stage, a finding also reported in breast cancer: Ogawa et al. (2008) reported that 73% (100/136) of AR-positive invasive mammary carcinomas were free of regional lymph node metastasis, compared to only 60% (49/81) of AR-negative breast cancers [50]. AR overexpression in FMCs was also negatively associated with the clinical stage at diagnosis, as in human breast cancer: according to Alshenawy et al. (2012), 91% (98/107) of AR-positive breast cancers were diagnosed at stages I–II, compared to 58% (25/43) of AR-negative carcinomas [44]. AR expression was negatively associated with the Elston and Ellis histological grades, including in triple-negative FMCs considered separately. This negative association between AR and histological grade has been reported in multiple publications on breast cancers, including in TNBCs alone [28, 64–66]. For example Tang et al. (2012) reported that 70% (77/111) of AR-negative triple-negative carcinomas were grade III while about 70% (11/16) of AR-positive triple-negative carcinomas were grade I–II [66]. And finally AR expression was negatively correlated with tumor-associated inflammation visible on HES-stained sections. Interestingly, tumor-associated inflammation was associated with poor prognosis in cats of the present study and in the veterinary literature [67]. A similar finding was recently reported in human breast cancer: according to Gujam et al. (2018), AR expression is associated with reduced tumor-associated inflammation [68]. We can conclude that as in human breast cancer, AR positivity in FMCs is mainly associated with favorable prognostic features: smaller pathologic tumor size, negative nodal stage, lower clinical stage, lower histological grade, absence of lymphovascular invasion, and lower tumor-associated inflammation.

FOXA1 positivity in FMCs was negatively associated with pathologic nodal stage, including in triple-negative FMCs, as reported in human breast cancer [56, 69, 70]. According to Albergaria et al. (2009) 31% (30/97) of FOXA1-positive breast cancers were pN+ compared to 69% (67/97) in FOXA1-negative carcinomas. However in ER-negative breast cancers, no association between FOXA1 and nodal stage was found [56]. FOXA1 was negatively associated with the clinical stage of FMCs, including triple-negative FMCs considered separately. Whereas in human breast cancer, no significant correlations were found between clinical stage and FOXA1 [54]. We also observed a negative correlation between FOXA1 and Elston and Ellis histological grade that was also observed in triple-negative FMCs. In human breast cancer, many studies have reported a negative association between FOXA1 and grade in consecutive series of breast cancers [56, 58, 71], but not in ER-negative breast cancers considered separately [56]. FOXA1 index ≥1% was also negatively correlated with lymphovascular invasion, including in triple-negative FMCs, as sometimes reported in breast cancer: Albergaria et al. (2009) found that 37% (30/81) of FOXA1-positive breast cancers were LVI+ compared to 55% (67/122) of FOXA1-negative carcinomas [56]. FOXA1 in our cohort was negatively associated with the Ki-67 proliferation index, in agreement with data reported in human breast cancer [52, 69]. And finally FOXA1 was positively associated with PR, AR and the luminal phenotype. Similarly, in the medical literature, most publications report a very strong correlation between FOXA1 positivity in breast cancers and the expression of the hormone receptors ER and PR [56, 57, 69, 72], confirmed by two meta-analyses [70, 73], AR [74], and the luminal-A phenotype [56, 57, 69, 72]. Although the positive correlation between FOXA1 and PR was also observed in our cohort, it is however interesting to note that FOXA1 and ER did not show significant association in cats of our cohort. This suggests a loss of the normal interplay between FOXA1 and ER [26] in feline mammary carcinomas. Loss of FOXA1 expression in ER+ breast cancers has been associated with resistance to endocrine therapy [69, 75, 76], thus ER+ FOXA1– FMCs in our cohort may not be hormone-responsive mammary carcinomas. Overall, we found that as in human breast cancer, FOXA1 positivity in FMCs was associated with favorable features such as negative nodal stage, lower clinical stage, lower histological grade and lower proliferation index. Most importantly FOXA1 was positively associated with AR, including in triple-negative FMCs.

The third objective of this study was to investigate the prognostic significance of AR and FOXA1 in FMCs, in order to evaluate their significance in the feline patient. AR overexpression was associated with longer disease-free interval, overall survival, and cancer-specific survival by univariate and multivariate survival analyses, independently of clinical stage at diagnosis (tumor size, lymph node metastasis, distant metastasis). AR overexpression was associated with favorable outcomes in both luminal and triple-negative FMCs. In human breast cancer also, AR overexpression is associated with better outcomes in patients with luminal [51, 77] and triple-negative carcinomas [66, 77–79]. In this study, FOXA1 index ≥1% was also associated with a longer disease-free interval, overall survival, and cancer-specific survival, by multivariate survival analyses, again with components of clinical stage as covariates of the models (tumor size, nodal status, distant metastasis). This finding was also observed in luminal but not in triple-negative FMCs. The significance of FOXA1 expression appears to be similar in human oncology, as FOXA1 positivity of breast carcinomas is associated with better outcomes especially in luminal carcinomas [52, 59, 72, 80]. By multivariate survival analysis, Hisamatsu et al. (2012) reported that FOXA1 was associated with longer relapse-free survival independently of the pathological size, histological grade and ER expression in luminal breast cancers [52]. In TNBC, FOXA1 expression alone was not associated with prognosis. However, Albergaria et al. (2009) reported a near significant result (*p* = 0.06) with the risk of disease progression being higher for FOXA1-negative TNBCs than for FOXA1+ TNBCs [56].

The fourth and last objective of our study was to better characterize AR-positive triple-negative FMCs in order to define a “luminal-AR” subtype. Indeed, gene expression studies have shown that TNBCs in women are a heterogeneous group, comprising a stable luminal-AR subtype, which has been reported by Lehmann et al. [20, 21], Burstein et al. [22], and Jézéquel et al. (referred to as Cluster 1 or C1 in their study) [23, 24]. Other groups have confirmed that the luminal-AR subtype is a distinct subtype of TNBC, characterized by high expression of the androgen receptor and enrichment in the hormone-regulated pathways that play an important role in steroid synthesis, and androgen/estrogen metabolism despite the absence of ER [81, 82]. Since transcriptome studies are expensive, Guiu et al. (2015) proposed a definition of the luminal-AR subtype using immunohistochemistry. The simple expression of AR is not enough to define a luminal-AR TNBC because AR-positive triple-negative carcinomas contain multiple subgroups with different behaviors and prognoses. FOXA proteins bind to DNA and induce rearrangements that facilitate DNA binding with other regulators such as steroid hormone receptors. In the healthy mammary epithelial cell, FOXA1 is necessary for the expression of ERα target genes [26]. In addition, preclinical studies suggest that FOXA1 allows AR to bind to ER DNA binding sites and thereby induce transcription of ER-related genes and thereby stimulate tumor proliferation [27]. Guiu et al. (2015) investigated whether AR and FOXA1 coexpression (evaluated by immunohistochemistry) could define the luminal-AR subtype of TNBC. In their study, 25.9% (126/487) of the triple-negative carcinomas expressed AR and 15.2% (70/460) were both AR- and FOXA1-positive, called luminal-AR [28]. This frequency was consistent with gene expression studies, in which 11 to 15.4% of triple-negative carcinomas were luminal-AR [20, 83]. Luminal-AR TNBCs defined by double positivity to AR and FOXA1 according to Guiu et al. were associated with a worse outcome than other TNBCs in terms of recurrence-free and overall survivals [84]. Other authors have used other immunohistochemical definitions of the luminal-AR subtype. For instance, Astvatsaturyan et al. (2018) defined luminal-AR TNBCs by their positivity to AR and negative expression of Epidermal Growth Factor Receptor (EGFR), a basal marker. In their study the luminal-AR subgroup (AR+ EGFR–, 9% of TNBCs) was associated with a better disease-free survival compared to other TNBCs [29]. In our current study, we decided to combine AR, FOXA1 and the basal marker CK14 in order to better characterize the luminal-AR subgroup of feline mammary carcinomas. And by doing so, we were able to find a non-basal like subgroup characterized by double positivity to AR and FOXA1 (AR+ FOXA1+ CK14–) that might represent the luminal-AR subtype of triple-negative FMCs, and a basal-like subgroup with AR positivity but loss of FOXA1 expression (AR+ FOXA1–CK14+). The non basal-like AR+ FOXA1+ CK14– subgroup (7/53 cases, i.e., 13% of AR+ triple-negative cases) was associated with a better outcome compared to the basal-like AR+ FOXA1–CK14+ subgroup by univariate and multivariate survival analyses. Inspired from the immunohistochemical definitions of luminal-AR TNBCs (AR+ FOXA1+ according to Guiu et al., AR+ EGFR– according to Astvatsaturyan et al.), we propose that luminal-AR triple-negative mammary carcinomas of cats can be defined by an AR+ FOXA1+ CK14– phenotype.

## Conclusion

In conclusion, we have shown that AR and FOXA1 were mainly associated with favorable features such as lower stage and lower histological grade, but also correlated together, as reported in human breast cancer, despite the fact that AR was more commonly expressed in FMCs than in breast cancers, and conversely FOXA1 was less frequently expressed in cats than in women with invasive mammary carcinomas. Both AR and FOXA1 were strong and favorable prognostic factors, independently of tumor stage at diagnosis. Within AR-positive triple-negative FMCs, we identified a non-basal AR+ FOXA1+ CK14– subgroup with AR and FOXA1 coexpression, which appears similar to the luminal-AR TNBC subgroup described in humans, and is associated with a favorable outcome. Female cats with invasive mammary carcinomas may thus be interesting spontaneous animal models to investigate new cancer therapy strategies such as anti-androgen or anti-androgen receptor molecules, especially in the aggressive and potentially non-hormone-responsive triple-negative basal-like AR+ FOXA1–CK14+ subgroup.

## Data Availability

The datasets used and analyzed during this study are available from the corresponding author on reasonable request.

## Abbreviations

AR: Androgen receptor
CK14: Cytokeratin 14
ER: Estrogen receptor alpha
FMC: Feline invasive mammary carcinoma
FOXA1: Forkhead box protein A1
HER2: Human epidermal growth factor receptor-2
HIER: Heat-induced epitope retrieval
HR: Hazard ratio
LVI: Lymphovascular invasion
pN: Pathologic nodal stage
PR: Progesterone receptor
pT: Pathologic tumor size
TNBC: Triple-negative breast cancer
